# A young lady with abdominal pain and facial lesions

**DOI:** 10.4103/0971-4065.70841

**Published:** 2010-07

**Authors:** M. E. Bhaskar, B. Kumar

**Affiliations:** Department of Internal Medicine, Sri Ramachandra Medical College and Research Institute, Porur, Chennai - 600 116, India

**Figure 1:**
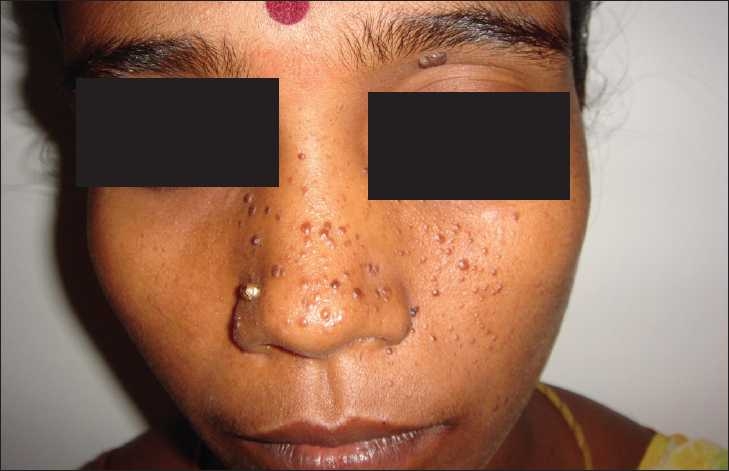
Cherry red papules on the face suggestive of adenoma sebaceum

**Figure 2:**
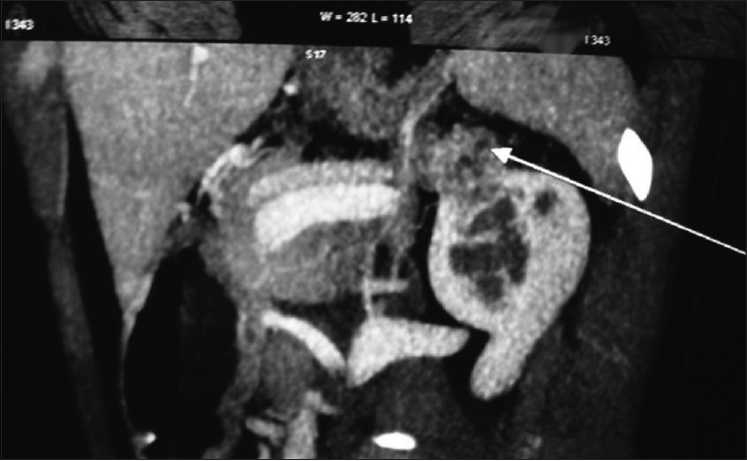
A reconstructed computerized tomography-abdomen film showing a mass lesion (arrow) in the upper pole of the left kidney with fat attenuation suggestive of renal angiomyolipoma

A 28-year-old female presented with abdominal pain for three months, which was constant with no specific character or aggravating factors. Her past was unremarkable except for multiple facial cherry red spots which were asymptomatic. She had completed high school and never had seizures. None of her family members had similar facial lesions. Physical examination revealed red colored papules over the face [Figure 1]. Rest of the examination was normal. Her baseline labs including renal function tests were normal. Computerized tomography (CT) of the abdomen showed a mass lesion in the upper pole of the left kidney with fat attenuation which was suggestive of angiomyolipoma [Figure 2]. CT-brain and echocardiography was normal. A diagnosis of tuberous sclerosis without neurological involvement complicated by symptomatic renal angiomyolipoma was made. In view of persistent abdominal pain the angiomyolipoma was treated with selective renal artery embolization. Her abdominal pain resolved in a week’s time. Follow-up screening, CT after eight weeks showed complete resolution of the angiomyolipoma.

Tuberous sclerosis is a common neuro-cutaneous syndrome characterized by the triad of adenoma sebaceum, seizures and mental retardation.[1] Adenoma sebaceum is present in most individuals with TS while the incidence of seizures and mental retardation is about 62 and 38% respectively.[2] Additional skin manifestations include ash-leaf spots, shagreen patch and ungual fibromas.[2] TS is a multi-system disorder and is associated with hamartomas and subependymal astrocytoma of the brain, retinal phakoma, cardiac rhabdomyoma and lymphangiomyomatosis of the lung.[1]

Renal lesions include angiomyolipoma, renal cyst and a marginal high incidence of renal cell carcinoma.[1] Pain is the most frequent symptom of renal angiomyolipoma, which may be complicated by life threatening hemorrhage. Renal angiomyolipoma greater than 4 cm should be angiographically studied and treated with partial nephrectomy or renal artery embolization to prevent the dreaded bleeding complication.[1] Adenoma sebaceum can be treated with cryosurgery or laser with good cosmetic results.[3,4]
